# Health service brokerage to improve primary care access for populations experiencing vulnerability or disadvantage: a systematic review and realist synthesis

**DOI:** 10.1186/s12913-019-4088-z

**Published:** 2019-04-29

**Authors:** Louise Thomas, Sharon Parker, Hyun Song, Nilakshi Gunatillaka, Grant Russell, Mark Harris, on behalf of the IMPACT Team, Grant Russell, Jeannie Haggerty, Jean-Frederic Levesque, Mark Harris, Simone Dahrouge, Virginia Lewis, Cathie Scott, Nigel Stocks

**Affiliations:** 1 Centre for Primary Health Care and Equity, University of New South Wales, Sydney, NSW 2052 Australia; 2 The Southern Academic Primary Care Research Unit, School of Primary Health Care, Monash University, Notting Hill, Victoria 3168 Australia

**Keywords:** Vulnerable populations, Health service brokerage, Primary care, Broker, Access to care, Realist evaluation, Systematic review, Candidacy theory, IMPACT study

## Abstract

**Background:**

Individuals experiencing disadvantage or marginalisation often face difficulty accessing primary health care. Overcoming access barriers is important for improving the health of these populations. Brokers can empower and enable people to access resources; however, their role in increasing access to health services has not been well-defined or researched in the literature. This review aims to identify whether a health service broker working with health and social service providers in the community can (a) identify individuals experiencing vulnerability who may benefit from improved access to quality primary care, and (b) link these individuals with an appropriate primary care provider for enduring, appropriate primary care.

**Methods:**

Six databases were searched for studies published between January 2008 and August 2015 that evaluated a health service broker intervention linking adults experiencing vulnerability to primary care. Relevant websites were also searched. Included studies were analysed using candidacy theory and a realist matrix was developed to identify mechanisms that may have contributed to changes in response to the interventions in different contexts.

**Results:**

Eleven studies were included in the review. Of the eight studies judged to provide detailed description of the programs, the interventions predominately addressed two domains of candidacy (identification of candidacy and navigation), with limited applicability to the third and fourth dimensions (permeability of services and appearances at health services). Six of the eight studies were judged to have successfully linked their target group to primary care. The majority of the interventions focused on assisting patients to reach services and did not look at ways that providers or health services could alter the way they deliver care to improve access.

**Conclusions:**

While specific mechanisms behind the interventions could not be identified, it is suggested that individual advocacy may be a key element in the success of these types of interventions. The interventions were found to address some dimensions of candidacy, with health service brokers able to help people to identify their need for care and to access, navigate and interact with services. More consideration should be given to the influence of providers on patient candidacy, rather than placing the onus on patients.

**Electronic supplementary material:**

The online version of this article (10.1186/s12913-019-4088-z) contains supplementary material, which is available to authorized users.

## Background

Many population groups experiencing vulnerability or disadvantage (such as people on a low income or from culturally and linguistically diverse groups) face difficulties in accessing primary health care, in Australia [1, 2] as well as in other developed countries [3–6]. Poor access to primary care translates into disparities in health status and outcomes [7], increased rates of hospitalisation for ambulatory care-sensitive conditions [8] and increased use of emergency departments [9]. One potential strategy to address the access barriers to primary health care for population groups experiencing vulnerability is through the use of brokers.

*Brokerage* is a concept rooted in sociology, where it is defined as “the process of connecting actors in systems of social, economic, or political relations in order to facilitate access to valued resources” [10]. A broker, by definition, is an individual who bridges a gap in social structure. Combined with the notion of social capital (information, support, and shared norms and values that flow through established social networks), brokers have the potential to empower and enable people to access and use a range of useful resources [11]. In primary care, brokers can potentially bridge the gap between individuals and essential health resources, including access to general practitioners (GPs). Competencies for conducting this type of role have been described in the literature [12]. Brokers can provide a range of instrumental and relational functions and processes to support patients to access primary care and directly identify providers willing to treat people experiencing vulnerability who require care [13]. Therefore, brokerage has particularly useful implications for individuals from disadvantaged and marginalised communities because it can enable increased (and more equitable) access to care. Previous studies have shown that addressing gaps in the accessibility of primary health care can attenuate negative health impacts arising from sociodemographic vulnerability (advanced age, ethnicity, low socioeconomic status and low literacy) and risk factors (e.g. smoking and obesity) prevalent among groups experiencing disadvantage [14, 15]. A 2013 study found that promoting primary care utilisation among groups experiencing vulnerability can be cost-effective [16].

A study of health service brokerage to link Aboriginal and Torres Strait Islanders in Australia with appropriate mainstream primary and allied health care found that brokers were able to successfully increase access to health services in the community by supporting patients to navigate their way through the healthcare system and directly identifying service providers willing to treat them [17]. However, despite their potential, the role of brokers/health service brokers is not well defined or researched in literature, including the mechanisms that have been used to link community living patients with primary care providers. To address this gap, we conducted a realist-informed systematic review which aimed to identify whether a health service broker working with health and social service providers in the community (either through personal interactions or the use of systems and processes) can (a) identify individuals experiencing vulnerability who are likely to benefit from improved access to quality primary care, and (b) link these individuals with an appropriate primary care provider for enduring, appropriate primary care. Results are reported for studies that evaluated a health service broker intervention linking adults experiencing vulnerability to primary care.

## Methods

### Context

This review forms part of the IMPACT (Innovative Models Promoting Access-to-Care Transformation) study, a five year Australian and Canadian research program with the overarching aim of transforming the primary health care organisational structure to improve access to appropriate care for vulnerable populations resulting in reduced unmet need, avoidable emergency department visits and avoidable hospitalisations. Integral to the IMPACT program of work is the establishment of, and engagement with, Local Innovation Partnerships (LIPs) which comprise decision makers, researchers, field workers and local community members who support the use of knowledge exchange and partnership practices. By identifying gaps in access to appropriate and equitable community-based primary health care in their own communities, each LIP aims to develop locally relevant innovations which address these gaps.

The research question was formulated in South Eastern Melbourne, Australia, where a broad group of stakeholders (policy and decision-makers, health and human service providers and community representatives) participated in two deliberative forums aimed at identifying priority primary care access gaps in South Eastern Melbourne and interventions which have the potential to address these gaps. The group elected ‘community worker’ as a broad innovation to explore. Building on these forums, and further consultations with key organisations in the region, clinicians and consumer advocacy groups, the broad ‘community worker’ intervention was refined to the health service broker model.

Our review followed standard systematic review methodology [18]. Realist synthesis was used to appraise the evidence, with the aim of explaining why particular interventions worked or did not work. It has been used to generate evidence on many primary care interventions, including interventions to improve equitable access to health care [19]. Reporting for this review followed the RAMESES publication standards for reporting realist synthesis [20] and the Preferred Reporting Items for Systematic Reviews and Meta-Analyses (PRISMA) statement [21].

### Definitions

#### Vulnerability

Vulnerability is relational and dynamic and arises from interactions between an individual’s characteristics and their environment [22]. We have referred to groups as experiencing ‘vulnerability’ in this study to express that certain groups within society are exposed to contextual conditions that place them more at risk than the rest of the population [23]. This follows a Social Determinants of Health approach, which recognises that the structural determinants and conditions of daily life lead to much of the health inequity between and within countries [24].

The groups that we focused on in this study were those whose demographic, geographic, economic and/or cultural characteristics may impede or compromise their access to community-based primary care services.

#### Brokerage

Brokerage was defined as a role or service to bridge the gap between individuals and access to appropriate primary care for groups experiencing vulnerability.

#### Primary care

Primary care is usually the first contact an individual has with the health system, and can include health promotion, prevention, early intervention, treatment of acute conditions, and management of chronic conditions [25]. These services can be delivered by a range of people, including general practitioners, nurses, allied health professionals, midwives, pharmacists, dentists, and Aboriginal health practitioners [26]. Settings in which primary care is delivered includes general practice, community health centres, allied health practices, and via communication technologies [26]. In this review, we also included hospital-based primary care clinics as these clinics can be accessed on an outpatient basis.

### Literature search

Comprehensive searches were conducted in Medline, Embase, All Evidence Based Medicine (EBM) reviews, CINAHL, PsychINFO and ProQuest. The search strategy was adapted for each database and included terms to encompass the types of role/worker conducting the intervention, as well as terms for the primary care setting and providers (see Additional file 1). An initial scoping review revealed that the term ‘health broker’ is not commonly used within the health care literature, thus a variety of different terms for the role were used in the search. The search was limited to empirical articles published in English, from OECD countries, between January 2008 and August 2015. Given the complexity around the concept of a ‘health service broker’, it was decided that this limited timeframe was necessary due to the extensive search which would be required to encompass all the terms used for this type of role.

Identified websites of interest from Australia, the United States of America, Canada and Europe were searched for relevant project or program evaluations. A list of possible documents for inclusion was compiled and these documents were reviewed in more depth by a second reviewer to determine relevance.

Comprehensive inclusion and exclusion criteria (Additional file 2) relating to the setting, population and intervention were developed based on the project definitions, scope of the review and knowledge gained through the deliberative forums about the key focus areas. The review was restricted to primary care settings in OECD countries (excluding primary care practices offering episodic care such as after hours or outpatient clinics) and to populations experiencing vulnerability.

Data was extracted to a template using Microsoft Access. This template was piloted using sample papers prior to data extraction commencing for the included studies. As part of the piloting process, two reviewers (LT and HS) extracted data separately for two studies. The reviewers then discussed the utility of the template, the clarity of the questions and the consistency of data extraction. The template was deemed suitable after undergoing this process. The template was designed to collect information on study type, country of origin, study location and publication details; inputs; description of the intervention, participants and setting; use of theory; dimensions of access addressed; and outcomes. Limited snowball sampling of reference lists was conducted during the data extraction phase. Relevant data from any additional publications identified for the included studies was also extracted.

### Quality assessment

Studies were assessed for their *rigour* (credible and trustworthy methods used to generate data) and their *relevance* (contribution to theory building and/or testing) using the process described by O’Campo [27] (see Additional file 3). This method was chosen in recognition of the fact that the most useful information for a realist evaluation may not necessarily be contained in studies that achieve the highest scores using traditional methods of quality assessment. Using the criteria shown in Additional file 3, one point was allocated for each positive response. For rigour, studies were classified as high (7 points), moderate (4–6) points or low (0–3 points). For relevance, studies were classified as ‘thick’ (i.e. containing detailed program description and discussion of implementation and reasons for study findings) (3–4 points) or ‘thin’ (i.e. lacking detailed program description and discussion of implementation and reasons for study findings (0–2 points). Scoring was conducted by one author (LT) and confirmed by a second author (HS). Studies were not excluded on the basis of this assessment, however, only the studies with high scores for relevance were used for the realist analysis given that these studies contained the most information on program components.

### Analysis

Our analysis utilised interpretive synthesis of the literature identified. To identify an overarching theory for the realist analysis, the studies were reviewed to extrapolate whether the development or implementation of any of the interventions were informed by theory. Candidacy theory, which describes “the ways in which people’s eligibility for medical attention and intervention is jointly negotiated between individuals and health services” [28] was selected as the overarching theory and the extent to which the dimensions of candidacy could be drawn out of the included studies was explored. In addition, the interventions were mapped via a realist matrix according to agency (whose actions are causing the change to occur), resources provided, and mechanisms (how the resources and the thing/person being changed interact) [29] in an attempt to get a deeper understanding of why the interventions worked, for whom and in what circumstances. Only studies that were assessed as “thick” (containing detailed program description and discussion of implementation and reasons for study findings) during the quality appraisal process (see Additional file 3) were included in this part of the analysis.

## Results

**Fig. 1:**
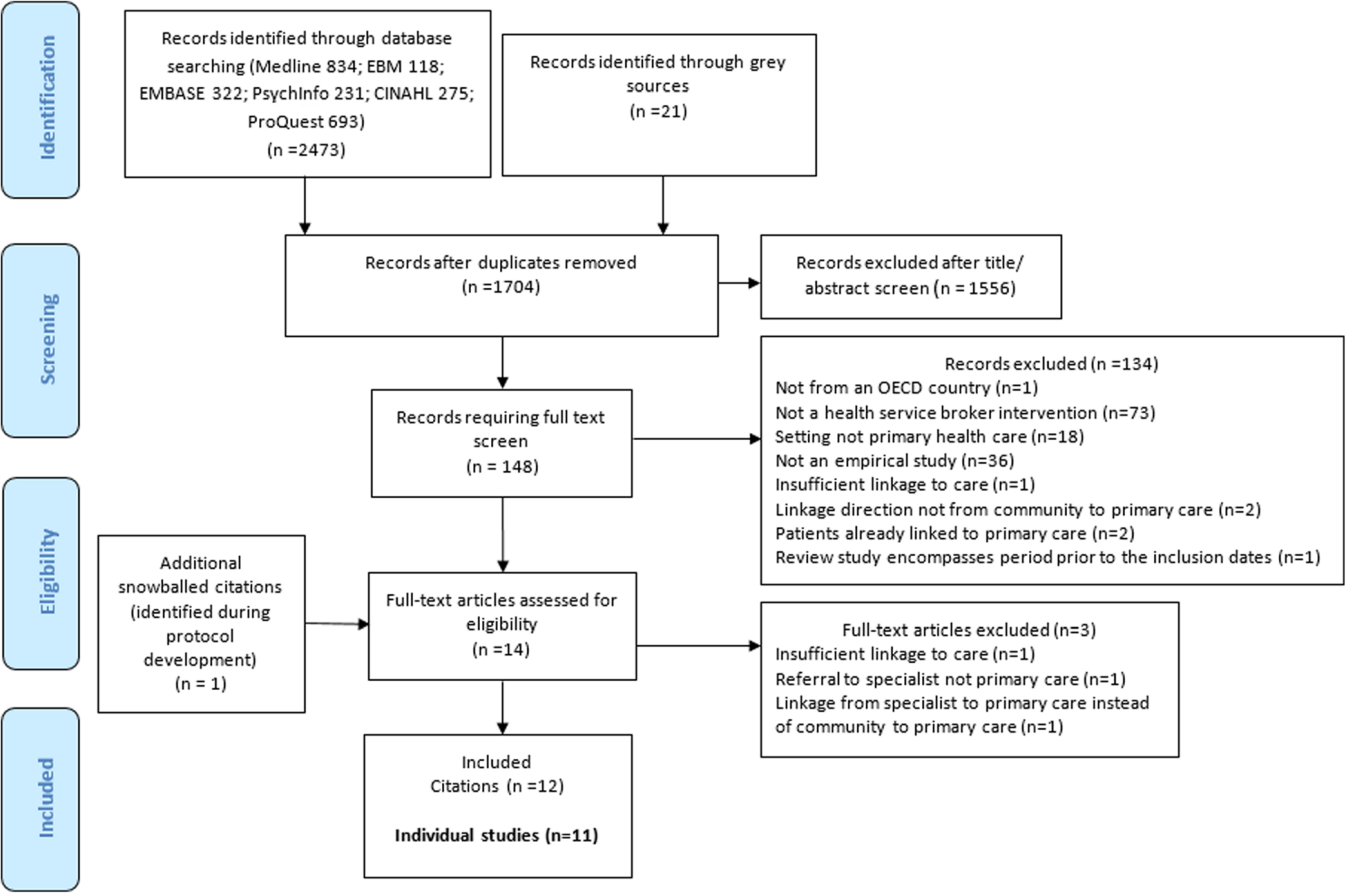
PRISMA flowchart of citation screening process

A total of 1704 citations were identified from the database searches after duplicates were removed. A total of 148 citations underwent a full text review to determine their eligibility, with a further 134 records excluded at this stage. Data extraction was conducted on 13 studies, however, an additional three citations were excluded during this process on the basis that the intervention had insufficient linkage to care (one citation), referral was to specialist rather than primary care (one citation) and linkage was from specialist to primary care rather than community to primary care (one citation). One citation identified was included in the final number of citations, however, data extraction was not conducted on this paper as it was an earlier publication for a study which was already included in our citations. An additional citation which was identified during the early stages of protocol development when scoping the term ‘health service broker’ was added, giving a final included number of 12citations. Eleven of these citations related to separate studies, although one study [30] built on the framework of another included study [31]. No relevant evaluative studies were identified from the grey literature. Full details of the results of the screening process are detailed in the PRISMA flowchart (Fig. 1).

### Characteristics of studies identified

The characteristics of the included studies are summarised in Table 1. A more detailed description of each study can be found in Table 2. Program evaluations were the predominant study type (*n* = 5). Apart from one study set in Australia, all of the studies were conducted in the United States. The primary care settings varied between community-based primary care clinic/provider (*n* = 3), hospital-based primary care clinic/provider (*n* = 4), public health clinics/agencies (*n* = 2) and community health centres (n = 4).

**Table 1:** Characteristics of the studies identified

Study characteristic		No of studies
Design	Randomised controlled trial	2
	Program evaluation	5
	Retrospective non-randomised cohort study	1
	Pre−/post-test intervention (no control)	1
	Quasi-experimental controlled community evaluation trial (pre- and post-test)	1
	Mixed methods	1
Primary care setting	Community-based primary care clinic/provider	3
	Hospital-based primary care clinic/provider	4
	Public health clinics/agencies	2
	Community health centres (safety net clinics/providers)	4
Intervention	Linking to primary care for screening for a condition	4
	Linking to primary care for general management	7
Vulnerability^a^	Older age	1
	Socioeconomic disadvantage	6
	Culturally and linguistically diverse group	7
	Indigenous (Aboriginal, first nations)	2
	Geographic disadvantage (rural)	2
	Disability/mental health issues	0
	Prison/prison leavers	2
	Healthcare-related disadvantage	10

^a^ some studies targeted multiple vulnerabilities and participants could fit into multiple groups

**Table 2:** Description of included studies

Study/Country † = associated citations	Stated aims	Study design	Vulnerability experienced	Outcomes assessed	Program quality (rigour) and program description (relevance)
Braun 2015 [36] United States	To present findings from an RCT of the use of navigators to reduce disparities that Asian and Pacific Islander Medicare recipients experience in accessing breast, cervical, prostate and colorectal cancer screening.	Randomised controlled trial	Asian and Pacific Islander Medicare beneficiaries with low screening participation on the medically underserved island of Moloka’i in Hawaii.	Screening prevalence, satisfaction with navigation services	Moderate (6) and thick (4)
Han 2009 [37] United States	To evaluate the effect of a lay health worker (LHW) intervention to promote breast cancer screening among Korean-American women who are predominantly first-generation immigrants	Pre−/post-test intervention (no control)	Korean-American women in the United States (predominately first-generation immigrants)	Changes in screening behaviours, changes in breast cancer knowledge and beliefs.	Moderate (4) and thick (4)
Hiatt 2008 [38] †^a^ United States	To evaluate an outreach intervention using lay health worker peers and a clinic provider inreach intervention to improve breast and cervical cancer screening	Quasi-experimental controlled community evaluation trial (pre- and post-test)	Underserved low-income African American, Chinese, Hispanic, and White women, 40 to 75 years of age, in two counties in the United States	Screening behaviour	Moderate (5) and thin (2)
Mason 2013 [34] United States	To present a process and outcome evaluation of a community patient navigation program to encourage mammography screening among underserved women.	Program evaluation	Underserved African American women in Georgia.	Mammogram uptake	Weak (3) and thick (4)
Dennis 2015 [17] Australia	To explore the views of Aboriginal people on their experiences of a brokerage model for access to community-based mainstream health services in an urban setting in New South Wales.	Mixed methods	Aboriginal people in South West Sydney	Improvement in access to health care, satisfaction with service	Moderate (4) and thin (2)
Findley 2011 [32] United States	To describe community-based care coordination programs for childhood asthma and operational statistics at five different sites	Program evaluation	Low income and ethnically diverse.	Caregiver confidence, change in health service utilisation (ED visits and hospitalisation), school absences, asthma management.	Moderate (5) and thick (4)
Johnson 2012 [39] United States	To describe the impact of community health workers (CHWs) providing community-based support services to enrollees who are high consumers of health resources in a Medicaid managed care system.	Retrospective non-randomised cohort study	High users of health services such as ED, people with high consumption of controlled substances, poorly controlled chronic diseases and people with high use of disease management referrals, family or provider referrals, and care coordination referrals.	ED utilization and payment; inpatient utilization and payment; prescription counts and payment; narcotic counts and payment; PCP visits and payment; specialist (non-PCP visits and payment).	Moderate (6) and thick (4)
Jordan 2013 [33] United States	To provide a) an overview of the NYC experience with HIV-infected people entering jails, b) a review of the methods used to provide services that facilitate continuity of care from jail to community primary care, and c) an assessment of the program outcomes of the transitional care coordination program.	Program evaluation	People with HIV returning home from jail	Releases to the community with a discharge plan, linkages to community primary care, linkages to primary care/releases to the community.	Moderate (5) and thick (3)
Krantz 2013 [30] United States	To evaluate whether a program to prevent coronary heart disease (CHD) with community health workers (CHWs) would improve CHD risk in public health and health care settings	Program evaluation	Residents in 34 primarily rural Colorado counties	Change in Framingham Risk Score (Primary) Changes in other health outcomes: Body mass index; Weight; Systolic BP; Diastolic BP; Total cholesterol; HDL cholesterol; LDL cholesterol (Secondary)	Moderate (4) and thick (4)
Wang 2012 [35] †^b^ United States	To compare two interventions designed to improve primary care engagement and reduce acute care utilization: Transitions Clinic, a primary care–based care management program with a community health worker, versus expedited primary care.	Randomised controlled trial	Individuals who were recently released from prison.	Visits to the study-assigned primary care clinic, visits to the medical or psychiatric ED that did not result in a hospitalization, rate of ED use, hospitalization, having any incarceration, time to first incarceration.	Moderate (6) and thick (4)
Whitley 2011 [31] United States	To describe the findings of a program employing community health workers to provide free CVD screening and education	Program evaluation	Underserved populations- including the uninsured, racial and ethnic minorities, the homeless, migrant and resort workers and small business employees	Referrals to primary care and/or other resources, follow-up contacts.	Moderate (4) and thin (2)

^a^Hiatt, R.A., et al., *Community-Based Cancer Screening for Underserved Women: Design and Baseline Findings from the Breast and Cervical Cancer Intervention Study.* Preventive Medicine, 2001. 33(3): p. 190–203

^b^ Wang EA, Hong CS, Samuels L, Shavit S, Sanders R, Kushel M. Transitions clinic: creating a community-based model of health care for recently released California prisoners. Public health reports (Washington, DC: 1974). 2010;125**:**171–7

### Study interventions

The interventions fell within two broad categories- those that linked participants to primary care for the purposes of screening cancer risk (n = 4), and those that linked to primary care for the purposes of general management (*n* = 7). Interventions targeted a wide range of groups experiencing vulnerability or multiple vulnerabilities. The most frequently targeted vulnerability was healthcare-related disadvantage (which included being medically underserved or uninsured, having overuse of ED, having poorly controlled or unscreened disease or risk factors, or untreated chronic disease) (*n* = 10) followed by cultural and linguistic diversity (n = 7).

### Study appraisal according to rigour and relevance

The appraisal scores for rigour and relevance of each study can be found in Table 2. No studies received the highest possible score of seven on the rigour scale, but three studies scored a six. The most common issues relating to rigour were that many of the studies did not provide details about determining sample size and did not include a comparison group. The majority of studies had a detailed program description and received the highest possible score of four on the relevance scale.

### Assessment of linkage to primary care

While linkage to primary care was not necessarily the primary outcome of all the included studies, all of the studies did involve either a directly quantifiable measure of the extent to which participants were linked to primary care [32–35] or an indirect measure of linkage to care such as changes in screening behaviour or referrals to care [17, 30, 31, 36–39]. Most of these measures were assessed using follow-up surveys or questionnaires. The majority of the studies (7/11) can be said to have been successful in linking their target population with primary care (Table 3).

**Table 3:** Measures of linkage and impact of studies on linkage outcomes

Study	Main linkage-related outcome	Direct or indirect measure of linkage to primary care	Measurement method	Impact on linkage?
Braun 2015 [36]	Change in cancer screening behaviours	Indirect	Baseline and exit surveys	Yes. Significant increase in screening. 57.0% had had a Papanicolaou test, 61.7% had had a mammogram, 54.4% had had a prostate-specific antigen test and 43% had had a flexible sigmoidoscopy or colonoscopy at the study exit. Specific number linked to primary care as a direct result of program not known
Dennis 2015 [17]	Access to mainstream health services	Indirect	Feedback from service users via semi-structured interviews, postal surveys and community forums	Yes. Feedback from surveys and qualitative comments suggested that brokerage service successfully linked Aboriginal Australians with primary care providers who were able to meet their needs. Specific number linked to primary care as a direct result of program not known
Han 2009 [37]	Change in breast screening behaviour	Indirect	Baseline and follow up questionnaires	Yes. Significant increase in screening. Screening increases at 6 month follow up were 31.9% for mammography, 23% for clinical breast examination and 36.2% for breast self-examination compared with baseline. Intervention involved referring participants to health providers but specific numbers seen by providers not given.
Hiatt 2008 [38]	Change in cancer screening behaviours	Indirect	Baseline and follow up surveys for random community samples (baseline and follow up groups were not the same)	No. No significant effects on screening behaviour between intervention and control groups. Specific linkage to primary care not measured.
Findley 2011 [32]	Number of participants obtaining an asthma action plan from a health professional during the intervention period	Direct	Baseline and follow up questionnaires	Yes. 76% of participants obtained an asthma action plan from a health professional at Los Angeles and New York site and 100% obtained an asthma action plan at Philadelphia site
Johnson 2012 [39]	Changes in number of office visits to primary care providers and specialists before, during and after the intervention period	Indirect	Utilisation data extracted from Molina Healthcare of New Mexico (Medicaid Managed Care provider organisation) records	Yes. CHWs assisted members to establish a primary care medical home- office visits to primary care providers and specialists dropped by half in non-CHW group but remained relatively stable in the CHW group. Specific number linked to primary care as a direct result of program not known
Jordan 2013 [33]	Number of participants who were linked to primary care in the community during intervention period	Direct	Transitional care coordinators contact community primary care provider to verify linkages to care and document this in the Electronic Health Record and monthly summary reports prepared with information on number linked to care	Yes. % of linkages to primary care/releases to the community: 70% in 2009, 75% in 2010 and 73% in 2011. The monthly average was 73% linked to primary care (monthly mean = 98).
Krantz 2013 [30]	Number of participants referred to medical or lifestyle resources during intervention period	Indirect	Number of referrals tracked through study database	Unclear. 53.5% of participants at risk for CHD received medical or lifestyle resource referrals. Specific number of these who made appointments with provider/s not known.
Mason 2013 [34]	Number of community members who had filled out a mammography interest/screening form who received a mammogram at the collaborating health facility in intervention period	Direct	Programmatic tracking forms used to keep track of mammogram appointment status	No. Only 21% of women who completed a mammography interest/screening form received mammograms at the collaborating health facility (a comparatively smaller proportion than other screening studies in the review)
Wang 2012 [35]	Number seen in primary care clinic at least once in the 12 month follow-up period and number seen 2 or more times	Direct	Utilisation data at follow-up extracted from the UCSF Clinical and Translational Science Institute’s The Health Records Electronic Data Set	Yes. More than 60% of participants were seen in primary care clinic at least once in the 12-month follow-up period, and 42% were seen 2 or more times. After 12 months of follow up, 37.7% of Transitions Clinic participants and 47.1% of Enhanced Primary Care participants made 2 or more visits to their assigned primary clinic.
Whitley 2011 [31]	Number referred to primary care and/or other resources during intervention period	Indirect	Number of referrals tracked through study database	Unclear. Over one-third of participants were referred to primary care and/or other resources. Specific number of these who made appointments with provider/s not known.

### Interpretive synthesis

To identify an overarching theory, the studies were reviewed to extrapolate whether the development or implementation of any of the interventions was informed by theory. The most commonly used theory was the Transtheoretical model, which was incorporated into three interventions [30, 37, 38]. The Transtheoretical model conceptualises behaviour change as involving progress through a series of six stages of change [40]. However, this model has been criticised for its presumptions of homogeneity and the arbitrary nature of the boundaries drawn between stages [41].

One study [17] utilised candidacy theory to examine the extent to which a brokerage service could increase access to health care. Candidacy is a construct developed by Dixon-Woods et al. [28] to conceptualise access to healthcare for groups experiencing vulnerability. The seven dimensions of candidacy (identification of candidacy, navigation, permeability of services, appearances at health services, adjudications, offers and resistance, and operating conditions and the local production of candidacy) [28] capture the complexity within the notion of access to health care and also recognise that the negotiation process of a person’s candidacy for care occurs within a broader environment context [42]. The candidacy construct emphasises that access is “highly dynamic and contingent, and subject to constant negotiation”, with candidacy being the “core organising construct” of access [28]. This negotiation process between individuals and professionals means that candidacy is constantly being expressed and re-expressed over time and space [43], with access representing the dynamic interplay between these processes [28]. The dimensions of candidacy are recursively interrelated and produced over both time and space [43].

Candidacy builds on the foundations of the Transtheoretical model but provides descriptions for healthcare experiences rather than stages and incorporates “the influence of professionals’ perceived manner, beliefs and competency on patient experience” [41]. As such, the candidacy framework can give a broader perspective of system and patient level factors influencing access to care, rather than solely focusing on patient factors. It is therefore very useful for understanding barriers and enablers to health care access [44]. Candidacy has been identified as a useful theoretical framework in a number of studies of disadvantaged populations and health care access [17, 28, 42–48].

### Dimensions of candidacy applicable to the included studies

The dimensions of candidacy from the eight studies with “thick” program description were extracted to the realist matrix (Table 4). Two dimensions of candidacy were addressed by the interventions (identification of candidacy and navigation), with some limited applicability to the third and fourth dimensions (permeability of services and appearances at health services).

**Table 4:** Realist matrix

Study	Agency	Context	Resources	Mechanisms	Outcome (anticipated change)
Screening interventions					
Braun 2015 [36]	Interaction between lay navigator and participant	Asian and Pacific Islander Medicare beneficiaries with low screening participation on the medically underserved island of Moloka’i in Hawaii. Bilingual LHWs from the community. They received training from an evidence-based navigator training program. Small geographic area made it easy for navigators to drive to people’s homes to transport to appointments or provide education.	Culturally compatible components- bilingual lay health workers. Navigators performed outreach, education, made appointments, sent reminders, provided transportation to appointments, communicated with providers and completed paperwork.	“Continuous outreach and education and finding elderly adults willing to “take a leap of faith to get screened” overcame reluctance of elderly adults to discuss cancer or participate in screening. That the majority of those screened did not have or get cancer helped convince others to try it.” P.369 **Dimensions of candidacy addressed:** ***Identification of candidacy; navigation; appearances at health services.***	Increased screening prevalence; satisfaction with navigation services
Han 2009 [37]	Interaction between lay health worker and participant	Korean-American women in the United States (predominately first-generation immigrants). Bilingual LHWs from the community. Received training and followed the project’s lay health worker manual designed to guide the in-class education and individualized follow up counselling.	2 h education sessions and individually tailored follow-up counselling via telephone or home visits (3–9 sessions) for 6 months. Culturally compatible components- bilingual lay health workers. Information provided on mammogram facilities near participants’ homes and low-income state cancer screening programs.	Interpersonally orientated method combining theory-based behavioural tailoring with use of culturally sensitive, trained LHWs addressed the particular issues often experienced by recent Korean immigrants. **Dimensions of candidacy addressed:** ***Identification of candidacy; navigation***	Improved screening behaviours, improved breast cancer knowledge and beliefs
Mason 2013 [34]	Interaction between community patient navigator and participant	Underserved African American women in Georgia. Patient navigators from community. Received training in breast cancer facts and statistics and how to deliver support and follow-up with community members while encouraging them to make and attend mammogram appointments.	Telephone follow-up delivered to encourage community members to make and keep their mammogram appointments (48 h after hosting a community event). Low-cost screening program. Literature distributed about importance of breast health.	Authors propose that patient navigator intervention addresses barriers to care through instruction and engagement of community member in understanding breast health and breast cancer combined with targeted follow up to encourage importance of receiving a mammogram. **Dimensions of candidacy addressed:** ***Identification of candidacy; navigation***	Increased mammogram uptake
General management interventions					
Findley 2011 [32]	Interaction between community health worker and participant	Low income and ethnically diverse group with poorly managed asthma. CHWs from the community. Trained to provide low literacy asthma education, environmental interventions and care coordination.	Culturally competent, tailored asthma education. Environmental interventions- home visits to reduce home environmental triggers. Referrals to health, educational, social and community resources. Regular follow up contact (home visits or telephone). Frequency varied by site. Asthma action plans.	Authors propose that care coordinators improved families’ ability to communicate with providers and provided follow-up to families regarding provider instructions. Skill mastery: Authors also propose that care coordinators may have reduced absences through “increased confidence in managing asthma amongst caregivers, leading to more comfort in sending a child to school despite a flare-up; better understanding of asthma and the need to help children stay involved in school and sports; and better day-to-day management of asthma.” P.60S **Dimensions of candidacy addressed: Identification of candidacy; navigation**	Increased caregiver confidence; change in health service utilisation (reduction in ED visits and hospitalisation); reduction in school absences; improved asthma management
Johnson 2012 [39]	Interaction between community health worker and participant	High users of health services such as ED, people with high consumption of controlled substances, poorly controlled chronic diseases and people with high use of disease management referrals, family or provider referrals, and care coordination referrals. Community health workers from the community. Received training in HIPAA laws/compliance, communication/learning styles, motivational interviewing, organizational skills, safety, community resources, behavioural health, personal growth.	Informational materials about community and clinical resources. Education, advocacy and social support.	None proposed or able to be extrapolated. **Dimensions of candidacy addressed:** ***Identification of candidacy; navigation; appearances at healthcare***	Decreased/more appropriate use of health services
Jordan 2013 [33]	Interaction between transition coordinator/patient care coordinator and participant	People with HIV returning home from jail. Transition coordinator/patient care coordinator from community. Received training in ‘warm transitions’ approach to linkages, motivational interviewing techniques and stages of engagement in care.	Discharge planning services addressing housing, food and clothing needs along with medical and social services. Transportation, accompaniment to primary care appointments and home visits. Culturally compatible components- bilingual transition coordinator/patient care coordinator. Discharge kit on release- medical summary, 7 day supply of medications and 21 day-prescriptions, condoms, health passport, listing of STD clinics and syringe exchange programs, a pocket guide to services designed for the criminal justice involved, and key words to use (“Jail Release Services”) when calling the NYC service telephone line. Motivational interviewing.	Empathy: “Caring, non-judgmental staff familiar with the needs of the population and the communities to which they return” p.S218 Face to face sessions beginning right away with pre-trial detainees. **Dimensions of candidacy addressed:** ***Identification of candidacy; navigation; permeability of services; appearances at health services***	Greater percentage of releases to the community with a discharge plan; increased linkages to primary care; greater percentage of linkages to the community/releases to the community.
Krantz 2013 [30]	Interaction between community health worker and participant	Residents in 34 primarily rural Colorado counties. Community health worker from the community. Undertook core curriculum components of a CHW certification program and also received additional coronary heart disease specific content expertise and formalized training in motivational interviewing techniques.	Health screenings and re-test screenings within 3–12 months after initial screening. Medical referrals and informational materials about community and clinical resources. Motivational interviewing to identify values and goals to promote healthy behaviour change. Follow up phone call after 2 weeks to check on status of referrals and action plans.	None proposed or able to be extrapolated. Authors note that the many embedded elements in the program make it difficult to isolate individual contributions of program components to improved outcomes. **Dimensions of candidacy addressed:** ***Identification of candidacy; navigation***	Change in Framingham Risk Score (reduction in risk), change in other health outcomes: reduced BMI, weight, systolic BP, diastolic BP and cholesterol levels.
Wang 2012 [35]	Interaction between community health worker and participant	Individuals who were recently released from prison. Community health worker from the community (with a personal history of incarceration). Received training on navigating the local health care and social service delivery system.	Case management (including referrals), chronic disease self-management support (including home visits) and health care navigation (including accompanying to appointments).	None proposed or able to be extrapolated. **Dimensions of candidacy addressed:** ***identification of candidacy; navigation; appearances at health services***	Increase in primary care utilisation, reduction in ED utilisation, difference in return to jail rates, reduction in number of hospitalisations. ED.

#### Identification of candidacy

Identification of candidacy relates to the ability of people to recognise their symptoms as needing medical attention or intervention [28]. All eight interventions involved lay workers who effectively took over this role from participants as a first step. The lay workers provided outreach to identify participants that needed to be linked with a health care service (screening or ongoing management) and provided a supportive, educational and advocacy arrangement with individuals that was designed to ‘lift’ or ‘activate’ their own candidacy. These types of interventions by their nature expose the participants to resources which aim to increase their understanding about their own health and healthcare needs. Through ongoing contact or follow up the individuals contemplate their need for continued care and develop the skills and knowledge to identify and act on this in the future.

#### Navigation

The navigation dimension of candidacy recognises that in order to find and utilise appropriate health services, people must firstly be aware that the service exists, as well as mobilise resources to help them get to that service [28]. All eight interventions contained components that promoted the notion of navigation. Primarily, personal interactions between the broker and the participant were the key to linking the participant to primary care or screening services. These interactions were focused on raising awareness of services (through referral, or by physically transporting the person to the service). In some cases, the lay worker ‘took up’ candidacy on behalf of the individual through activities such as contacting providers to arrange appointments for the participant [33–37, 39]. In other situations, these resources may have facilitated the development of an individual’s own candidacy, such as through increased health literacy [39]. Developing the individual’s own capacity to navigate the system as opposed to the lay worker navigating the system on their behalf may have a greater impact in the longer term. However, this is difficult to assess in the studies as the interventions generally involved the provision of both types of resources- ones that may have contributed to the development of the individual’s own capacity, as well as ones that involved the lay worker utilising their own capacity on behalf of the individual.

#### Permeability of services

Permeability of services refers to the ease with which people can use services [28]. Porous services require few qualifications of candidacy and the mobilisation of relatively few resources [28]. Permeability of services can be promoted through factors such as providing transport services, having flexible appointment structures, minimal out-of-pocket expenses and welcoming physical spaces [44]. Only one study [33] had capacity building aspects that may have contributed to increased service permeability. This study encouraged community health centres to “create, expand and enhance clinic hours where appointments are not required” [33]. However, the uptake or effect of this was not discussed.

#### Appearances at health services

The fourth dimension of candidacy, appearances at health services, recognises that in order to receive appropriate services, service users have to assert their needs [41]. This requires competencies such as the ability to understand and explain the issue [28] and could therefore be challenging for people experiencing vulnerability. Four interventions incorporated aspects relating to this dimension of candidacy. In some of the interventions, the lay worker accompanied individuals to their appointments and assisted in their interactions with the health care provider [33, 35, 36, 39]. By facilitating communication between the patient and the provider, the lay worker may have assisted the patient to assert their needs. However, assisting an individual to assert their claim may help them to progress through the system the first time, but may not have any residual effects for any future appearances at health services or serve to build the capacity of the individual. The short follow-up periods of the studies limited our ability to explore this issue within the review.

Six of the eight studies included in the realist analysis were judged to have successfully linked their target group to some form of primary care, using either directly quantifiable measures or indirect measures of linkage to care (as outlined earlier and shown in Table 4 for all included studies).

Of the studies which did not result in improved linkage to primary care [30, 31, 34, 38] (shown in Table 3), only two studies [30, 34] had significantly “thick” program descriptions to be included in the realist matrix. Both of these studies only addressed two dimensions of candidacy. However, we are unable to conclude that more than two dimensions are required to be addressed for an intervention to be successful, as both the Findley [32] and Han [37] studies also addressed only the first two dimensions of candidacy but did have a positive impact on linkage to primary care.

## Discussion

Candidacy was found to be a useful way to conceptualise the influence of health service brokers on access to health care. Health service brokers were able to influence some dimensions of candidacy for individuals experiencing vulnerability, including identifying that they are a priority and eligible for health care, and the types of health care that might be appropriate. The majority of the studies were able to successfully link individuals experiencing vulnerability to primary care, which suggests that interventions which address aspects of candidacy can positively influence the journey and interactions of individuals experiencing vulnerability with health services and providers to ultimately improve access to health care. While we were not able to identify for which groups this might work better, we were able to identify that advocacy on behalf of the individual until the participant has been supported to be able to recognise and negotiate their own needs for healthcare may be a key element in the success of these types of interventions. It should be noted that while brokering primary care may increase access to health services, it is not a complete solution to health inequity. Tackling health inequity in a holistic way requires taking action on the social determinants of health [49], however, this is beyond the scope of this review.

Many of the studies identified through the review either directly stated or implied that advocacy by individuals, such as community health workers or lay health workers, was a key part of the intervention. The Community Preventive Services Task Force findings from a recent review of interventions that used community health workers to prevent cardiovascular disease in high risk populations emphasised the unique position of community health workers as trusted community members to advocate on behalf of individuals and communities and help build capacity [50]. Interventions utilising professional staff rather than community members should consider the implications of this for the advocacy component of the role. Relationships may need to be built over time to establish trust with the community. Further, attention should be given to the reporting lines and organisational embeddedness of the broker as this may enhance or constrain their ability to advocate on behalf of the patient.

The lack of attention given to the patient/provider interactions in the study is probably a reflection of the limited scope of the studies, but could be a criticism of the interventions as they appear to have been driven by an inherent assumption that once people had been assisted to access a provider, that they would then continue to seek or receive the care that they needed from this provider. In reality, this may not be the case as the identification of need by a provider does not necessarily mean that people will also recognise (or prioritise) this need and utilise the services available, or that they will receive appropriate care from this provider and/or service. Resistance (both active and passive) has implications for service provision and interventions should include measures to address resistance, such as incorporating follow-up and ongoing contact with individuals into the intervention design.

The influence of providers on patient candidacy should not be overlooked. The fact that not many of the studies discussed system level changes or capacity building activities to improve permeability of health services suggests that the majority of the interventions are focused on only one side of the problem and as such, are likely to be short term fixes only. These interventions place the onus on the individual patient to change or work harder to reach the service, when in fact the providers themselves might need to increase the permeability of services in order to allow more people to navigate through the system, without the need for interventions. The Access framework by Levesque et al. [51] also supports the need to look beyond patient factors alone to overcome issues of access to health care. The Access framework describes access as being two-sided, with service level factors interacting with corresponding abilities of persons, along a healthcare continuum to generate access. While Candidacy theory [28] recognises the influence of the factors beyond the individual (e.g. the setting and environment in which care takes place), it does not clearly delineate these into two ‘sides’. Future interventions should aim to address both patient and service level factors in order to improve access as this review has identified that there is an overemphasis on user responsibility compared to service responsibility. For example, services should be tailored to the community to enable the influence of the local environment on candidacy to be recognised and addressed, rather than utilising a “one size fits all approach”. The Australian National Safety and Quality Health Service Standards (second edition) [52] strongly emphasise the importance of partnering with consumers to plan, design, deliver, measure and evaluate systems and services. Primary care services that are not currently “permeable” to patients experiencing vulnerability should take steps to break down these barriers by engaging with the community and learning from their lived experiences.

### Review limitations

The generalisability of results is limited by the small number of studies identified in the review and the varying methodological quality of the studies. Although a realist approach was utilised to try and identify the mechanisms behind the interventions, many studies, even those providing qualitative data, were largely unable to shed light on this. This issue has been reported by other authors conducting realist reviews- a review of published realist reviews identified that the authors of these publications frequently reported that they were unable to identify details on the mechanisms by which an intervention was expected to work [53]. The scope of the review had to be widened as it could not be determined whether participants in the studies were linked to an enduring form of primary care or whether contact with providers was a one-off, given that the majority of studies had short follow-up periods of less than 6 months. As a result, some of the included interventions had more of a focus on linking to primary care so that participants were adherent to screening recommendations, rather than on linking participants to a regular form of primary care to meet a wider range of health needs. More evidence is needed as to whether positive intervention effects can be sustained, as the studies included in this review generally conducted follow-up for a period of 6 months or less from the end of the intervention.

## Conclusions

Specific mechanisms that may have contributed to changes in response to the interventions in the different contexts were not able to be identified, although advocacy may be a key element in the success of these types of interventions. The majority of the studies were able to link individuals experiencing vulnerability to primary care and the interventions addressed several dimensions of candidacy. In particular, the brokers helped people to identify their need for care and to access and navigate services.

## Additional files

Additional file 1: Search strategy adapted for each database. (DOCX 16 kb)

Additional file 2: Inclusion and exclusion criteria. (DOCX 20 kb)

Additional file 3: Quality assessment. (DOCX 17 kb)

## Abbreviations

GPs: General practitioners
IMPACT: Innovative Models Promoting Access-to-Care Transformation
LIPs: Local Innovation Partners
