# Supplementary material for: Health service brokerage to improve primary care access for populations experiencing vulnerability or disadvantage: a systematic review and realist synthesis
Source: BMC Health Serv Res. 2019 Apr 29;19:269. doi: 10.1186/s12913-019-4088-z (PMC6489346; doi:10.1186/s12913-019-4088-z)
Supplement: Supplementary file 1 — Search strategy adapted for each database. (DOCX 16 kb) [file 12913_2019_4088_MOESM1_ESM.docx]

## Additional file 1: Search strategy adapted for each database

|  | MEDLINE | EMBASE | PsycInfo | ALL EBM | CINAHL | ProQuest |
| --- | --- | --- | --- | --- | --- | --- |
| 1 | community health worker*.tw | community health worker*.ti | community health worker*.tw | community health worker*.tw | TI “community health worker#” | (ti(community health worker*) |
| 2 | lay health worker*.mp | lay health worker*.mp | lay health worker*.mp | lay health worker*.mp | “lay health worker#” | all(lay health worker*) |
| 3 | lay worker*.mp | lay worker*.mp | lay worker*.mp | lay worker*.mp | “lay worker#” | all(lay worker*) |
| 4 | community health representative*.mp | community health representative*.mp | community health representative*.mp | community health representative*.mp | “community health representative#” | all(community health representative*) |
| 5 | community health advocate*.mp | community health advocate*.mp | community health advocate*.mp | community health advocate*.mp | “community health advocate#” | all(community health advocate*) |
| 6 | health broker*.mp | health broker*.mp | health broker*.mp | health broker*.mp | “health broker*” | all(health broker*) |
| 7 | cultur* broker*.mp | cultur* broker*.mp | cultur* broker*.mp | cultur* broker*.mp | “cultur* broker*” | all(cultur* broker*) |
| 8 | link worker*.mp | link worker*.mp | link worker*.mp | link worker*.mp | “link worker#” | all(link worker*) |
| 9 | liaison worker*.mp | liaison worker*.mp | liaison worker*.mp | liaison worker*.mp | “liaison worker#” | all(liaison worker*) |
| 10 | care co*ordinator*.mp | care co*ordinator*.mp | care co*ordinator*.mp | care co*ordinator*.mp | “care co#ordinator#” | all(care co*ordinator*) |
| 11 | indigenous health worker*.mp | indigenous health worker*.mp | indigenous health worker*.mp | indigenous health worker*.mp | “indigenous health worker#” | OR all(indigenous health worker*) |
| 12 | patient navigator*.mp | patient navigator*.mp | patient navigator*.mp | patient navigator*.mp | “patient navigator#” | all(patient navigator*) |
| 13 | patient advocate*.mp | patient advocate*.mp | patient advocate*.mp | patient advocate*.mp | “patient advocate#” | all(patient advocate*) |
| 14 | peer navigator*.mp | peer navigator*.mp | peer navigator*.mp | peer navigator*.mp | “peer navigator#” | all(peer navigator*) |
| 15 | lay navigator*.mp | lay navigator*.mp | lay navigator*.mp | lay navigator*.mp | “lay navigator#” | all(lay navigator*) |
| 16 | health liaison*.mp | health liaison*.mp | health liaison*.mp | health liaison*.mp | “health liaison#” | all(health liaison*) |
| 17 | 1 or 2 or 3 or 4 or 5 or 6 or 7 or 8 or 9 or 10 or 11 or 12 or 13 or 14 or 15 or 16 | 1 or 2 or 3 or 4 or 5 or 6 or 7 or 8 or 9 or 10 or 11 or 12 or 13 or 14 or 15 or 16 | 1 or 2 or 3 or 4 or 5 or 6 or 7 or 8 or 9 or 10 or 11 or 12 or 13 or 14 or 15 or 16 | 1 or 2 or 3 or 4 or 5 or 6 or 7 or 8 or 9 or 10 or 11 or 12 or 13 or 14 or 15 or 16 | S1 or S2 or S3 or S4 or S5 or S6 or S7 or S8 or S9 or S10 or S11 or S12 or S13 or S14 or S15 or S16 | 1 or 2 or 3 or 4 or 5 or 6 or 7 or 8 or 9 or 10 or 11 or 12 or 13 or 14 or 15 or 16 |
| 18 | exp Primary Health Care | exp Primary Health Care | exp Primary Health Care | exp Primary Health Care | MH “Primary Health Care” | (MJMESH.EXACT("Primary Health Care") |
| 19 | exp Family Practice | Family practice.mp | exp Family Medicine | exp Family Practice | MH “Family Practice” | MJMESH.EXACT("Family Practice") |
| 20 | exp. General Practice | exp General Practice | family practice.mp | exp. General Practice | “general practice” | MJMESH.EXACT("General Practice") |
| 21 | exp. Community Health Services | exp. Community care | general practice.mp | exp. Community Health Services | MH “Community Health Services+” (exp) | MJMESH.EXACT("Community Health Services") |
| 22 | exp. Social Welfare | exp. Social Welfare | exp. Community Services | exp. Social Welfare | MH “Social Welfare+” (exp) | MJMESH.EXACT("Social Welfare") |
| 23 | 18 or 19 or 20 or 21 or 22 | 18 or 19 or 20 or 21 or 22 | community health services.mp | 18 or 19 or 20 or 21 or 22 | S18 or S19 or S20 or S21 or S22 | 18 or 19 or 20 or 21 or 22 |
| 24 | 17 and 23 | 17 and 23 | exp. Social Services | 17 and 23 | S17 and S23 | 17 and 23 |
| 25 | Developing countries | Developing countries | 18 or 19 or 20 or 21 or 22 or 23 or 24 | Developing countries | MH “Developing Countries” | MJMESH.EXACT("Developing Countries")) |
| 26 | 24 not 25 | 24 not 25 | 17 and 25 | 24 not 25 | S24 not S25 | 24 not 25 |
| 27 | editorial.pt | editorial.pt | Developing countries | editorial.pt | PT editorial | at.exact("Letter To The Editor" OR "Editorial" OR "Commentary") |
| 28 | letter.pt | conference abstract.pt | 26 not 27 | letter.pt | PT letter | 26 not 27 |
| 29 | comment.pt | letter.pt | editorial.dt | comment.pt | PT commentary | Limit 28 to la.exact("English")) |
| 30 | 27 or 28 or 29 | 27 or 28 or 29 | letter.dt | 27 or 28 or 29 | S27 or S28 or S29 | Limit 29 to yr= 2008-03/08/2015 |
| 31 | 26 not 30 | 26 not 30 | abstract collection.dt | 26 not 30 | S26 not S30 |  |
| 32 | Limit 31 to (English language and yr= “2008- Current”) | Limit 31 to (English language and yr= “2008- Current”) | 29 or 30 or 31 | Limit 31 to (English language and yr= “2008- Current”) | S26 not S30. Limiters- Date published: 20080101-20151231; English |  |
| 33 |  |  | 28 not 32 |  |  |  |
| 34 |  |  | Limit 33 to (English language and yr= “2008- Current”) |  |  |  |

Note: Database searches were run on 3/08/15, with the exception of ProQuest, which was run on 4/06/18 (but covered the same time period as the other searches).
