# Supplementary material for: Health service brokerage to improve primary care access for populations experiencing vulnerability or disadvantage: a systematic review and realist synthesis
Source: BMC Health Serv Res. 2019 Apr 29;19:269. doi: 10.1186/s12913-019-4088-z (PMC6489346; doi:10.1186/s12913-019-4088-z)
Supplement: Supplementary file 2 — Inclusion and exclusion criteria. (DOCX 20 kb) [file 12913_2019_4088_MOESM2_ESM.docx]

## Additional file 2. Inclusion and exclusion criteria

| **Study characteristics** | **Inclusion** | **Exclusion** |
| --- | --- | --- |
| Intervention | **Delivery by person:**   - A person of any discipline - No specific training requirement but good knowledge of local area - Does not provide direct medical treatment (although could have a background in health or social services). Can have a dual role (health practitioner and health broker) - May have a dual role providing health care in addition to a brokerage role however, their main task is the provision of connectivity between social services, clients attending social services and general practice- establishing links between services through relationship, trust and rapport building - Possesses broad knowledge of health and social service sectors. - Broker contact with patient is primarily supportive but also facilitates access to primary care - Broker contact with patient and provider is generally ongoing in nature, but we will include one-off contact for its relevance to resource-poor areas - Capacity building role in one or more sectors through education, introduction of new systems, or work practices | **Delivery by person:**   - Provides direct medical treatment and doesn’t have a dual role providing medical care *and* brokerage activities. We will however include if they are providing basic screening activities such as blood pressure monitoring, weight/BMI, administering questionnaires, taking temperatures. |
|  | **Additional resource/system utilised:**   - Eg. Identification of vulnerability through use of index or other screening tools; database or register to help link person to appropriate primary health care (PHC) service |  |
| Setting | OECD countries. | Non-OECD countries |
|  | Primary health care:   - Family/general practices - Community health centres - Primary health clinics - Hospitals - general practice - Aboriginal health care centres - Migrant health services - Refugee health centres   Social services:  Within the framework of this review, social services are various community-based services delivered by welfare organisations supporting the physical (non-medical), social, emotional, spiritual and financial wellbeing of vulnerable communities. These services can be funded and/or provided by governmental (local, state/territorial, national) and non-governmental (private or public agencies, NGOs) organisations. Examples include:   - front-line community agencies (such as migrant services (e.g. Gippsland Multicultural Services, Spectrum Migrant Resource Centre), housing services (e.g. The Salvation Army Red Shield Housing Victoria), disability services (e.g. House with No Steps) etc. - national peak organisations of consumers and service providers (e.g. Australian Council of Social Service, Federation of Community Legal Centres,) - Welfare agencies (e.g. Department of Human Services) - secular and religious welfare agencies (e.g. Salvation Army, Mission Australia); and, - low income consumer groups and unions (e.g. Consumer Utilities Advocacy Centre)   (Adapted from Australian Council of Social Service [38]) | - Tertiary setting. - Primary care practices offering only episodic care (i.e. after hours, ambulatory/outpatient clinics. |
| Outcomes | Short-term:  Patient:   - Attends PHC appointment - Can identify a regular source of primary care (or consistent avenue for care) - Satisfaction with care provided by GP and/or broker - Improved self-efficacy (measured by survey or questionnaire or qualitative interview)   Health and social welfare workers:   - Collaborate/work well with health service broker (measured by a satisfaction survey or questionnaire, broker log/diary, meeting with health and social welfare workers- qualitative interview) - Utilise available database for appropriate referral to allied/specialist health services. - Report having successfully linked clients with broker   PHC providers:   - Report having more clients and contact with clients who are vulnerable by certain demographic characteristics. - Satisfaction with the process - Workload   Broker:   - Greater skill set through training, if provided (satisfaction with skills training and/or satisfaction with ability to carry out role), and job security   Medium-term:  Patient:   - Returns for follow-up appointment within 6-12 months.   Health and social welfare workers:   - Report having fewer clients “on their books” with health needs that continue to be unaddressed.   PHC providers:   - Report improvement or better management of health needs; greater participation in preventative care of vulnerable clients (less likely to have acute needs)   Long-term:   - Number of vulnerable persons linked from community settings to the GP (over 12 months) - Patient and GP maintain contact (satisfied with care) - Reduction in avoidable ED presentations and hospitalisations by vulnerable communities. - Service agreements with partner organisations continue beyond initial 12 months.   Resources and capacity:   - The use of resources: increased knowledge of existing GPs, appropriateness of GP (language, culture, location, expertise), patient profiles. Implementation of tool on either side (general practice or social services) that facilitates interaction - Capacity building at both a system and patient level: Changing practices/systems to identify vulnerable populations and incorporate their needs (e.g. Incorporating interpreter, expanding network - Identification of mechanisms (potential system level changes/redesign opportunities) to build capacity and improve access |  |
| Population | Patient from a vulnerable population group (CALD communities- recent immigrants, recently arrived refugees, asylum seekers; Aboriginal- indigenous, first nations; socioeconomic disadvantage- unemployed/underemployed, low income, public housing, homeless; geographic disadvantage- socially isolated, public transport inaccessibility, rural/remote areas; disabilities; mental health issues). |  |
| Other | Written in English. |  |
|  | 2008-current |  |
