# Supplementary material for: Health service brokerage to improve primary care access for populations experiencing vulnerability or disadvantage: a systematic review and realist synthesis
Source: BMC Health Serv Res. 2019 Apr 29;19:269. doi: 10.1186/s12913-019-4088-z (PMC6489346; doi:10.1186/s12913-019-4088-z)
Supplement: Supplementary file 3 — Quality assessment. (DOCX 17 kb) [file 12913_2019_4088_MOESM3_ESM.docx]

## Additional file 3. Quality assessment

The quality appraisal method used has been described in detail by O’Campo [19]. The following questions relating to rigour were applied to each of the included studies:

*Is there a clear statement of the aims of the research?*

*Did the study include an appropriate comparison group?*

*Did the study use appropriate eligibility criteria to obtain its target group?*

*Did the study use standardized methods for selecting/putting people into the study and state how they did this?*

*Did the study provide details about determining sample size?*

*Did the study have a comparatively long study period (≥6 months)?*

*Is the methodology appropriate for what they were trying to achieve?*

One point was allocated for each positive response and studies were graded as high quality (7 points), moderate quality (4-6 points) or of weak quality (0-3 points). Studies were not excluded on the basis of this assessment of rigour, however, the scores were used to compare efficacy across the high and moderate quality studies.

The following questions were applied to assess relevance within the studies:

*Is the intervention program description detailed?*

*Did the study describe factors that affected program implementation?*

*Did the study consider reasons for the results that they achieved?*

*Did the study discuss reasons for program success or failure?*

One point was allocated for each positive response and studies were graded as ‘thick’ (3-4 points) or ‘thin’ (0-2 points). Again, studies were not excluded on the basis of this assessment, however, only the thick studies were used for the realist analysis given that these studies contained the most information on program components.
